# The complete mitochondrial genome of the southern calanoid copepod *Calanus simillimus* Giesbrecht, 1902

**DOI:** 10.1080/23802359.2022.2093678

**Published:** 2022-07-07

**Authors:** Irina Smolina, Marina Espinasse, Cesilie Røtnes Amundsen, Boris Espinasse

**Affiliations:** a Faculty of Biosciences and Aquaculture, Nord University, Bodø, Norway; b Ecosystem processes Research Group, Institute of Marine Research, Tromsø, Norway; c Department of Arctic and Marine Biology, UiT The Arctic University of Norway, Tromsø, Norway

**Keywords:** *Calanus simillimus*, mitochondrial genome, calanoid copepod, phylogenetic analysis

## Abstract

The complete mitochondrial genome of *Calanus simillimus* is 27,876 bp in length (GenBank accession OK500294) and containing 13 protein-coding genes (PCGs), 2 rRNA genes, 22 transfer RNA genes. The gene order is novel compared to other *Calanus* species and copepods with sequenced mitogenomes. Phylogenetic analysis suggests that *C. simillimus* represent a fourth group within *Calanus* genus in addition to *C. hyperboreus, C. finmarchicus* and *C. helgolandicus* groups. The complete mitochondrial genome of *C. simillimus* will be useful for species identification, population genetics, phylogenetic and evolutionary studies among copepods.

*Calanus simillimus* Giesbrecht, 1902 is one of the abundant calanoid species occurring in the Southern Ocean and associated subpolar areas. Like other calanoid species, it stores energy during the productive season and later uses it to survive through winter (Atkinson 1991). As a lipid rich species, it represents a valuable and important source of food for marine predator species, including marine mammals, sea birds and fish (Ducklow et al. 2007). Despite its ecological importance, genomic resources for this species are very scarce and limited to several partial sequences of cytochrome oxidase subunit 1 (CO1) gene and 28S ribosomal RNA gene. Reshuffled gene order in mitogenome is a known feature in calanoids and copepods (e.g. Choi et al. 2019), however up to date only several species of *Calanus* genus shown in addition a presence of several long non-coding sequences (Weydmann et al. 2017). Therefore, the complete mitochondrial genome of *C. simillimus* will be useful for species identification, population genetics and phylogenetic studies in *Calanus* genus and Copepoda, as well as evolutionary studies on appearance and role of long non-coding elements in mitogenomes.

Zooplankton samples were collect during cruise MOBYDICK south of The Kerguelen Islands (lat: 50.62S; long 72.00E) in March 2018 by WP2 net and preserved right away in ethanol. Individuals of *C. simillimus* were morphologically identified using the reported literature (Razouls 1994). DNA was extracted from a single individual using DNeasy Blood and Tissue kit (Qiagen) and 300 ng were used as library input. Remaining DNA and voucher specimens were deposited at Faculty of Biosciences and Aquaculture, Nord university (DNA accession number: Csim_4, collection code M2-WP2-13.03.18, contact Irina Smolina, irina.smolina@nord.no). Genomic DNA was sonicated to 350 bp fragment length peak using a Covaris S220 (Covaris) and further prepared using NEBNext Ultra II DNA Library Prep Kit (New England Biolabs) and consequently sequenced using Illumina NextSeq 500 platform (Illumina, USA).

Resulting 52 millions pair-end raw reads were used for de novo assembly using SPAdes v3.13.0 (Bankevich et al. 2012) with K-mer auto. The longest obtained contig was blasted against nucleotide database at NCBI data base and resulted in significant hits to mitochondrial genes of calanoid copepods. The contig was inspected for terminal repeats to evaluate its circularity and completeness leading to conclusion that obtained mitochondrial contig is complete and circular. Further, the contig was annotated using web server MITOS2 with RefSeq 63 Metazoa database and invertebrate genetic code (Donath et al. 2019). Gene rearrangements were explored using CREx (Bernt et al. 2007).

The complete mitogenome of *C. simillimus* (GenBank accession OK500294) is 27,876 bp in length and includes a standard set of 22 transfer RNAs (tRNAs), 2 ribosomal RNAs (rRNAs), 13 protein-coding genes (PCGs) and three large (over 1,5 Kb) non-coding regions. Order of mitochondrial genes in *C. simillimus* is novel compared to other *Calanus* species and copepods. In relation to the pan-crustacean basal pattern (Kim et al. 2013) the gene rearrangements in *C. simillimus* include several gene reversals, transpositions and a tandem duplication random loss event. The frequency of nucleotides is A: 35.3%, T: 34.3%, G: 15.0% and C: 15.4% with a GC content of 30.4%.

The 13 identified PCGs vary in length from 162 (ATP8) to 1,713 bp (NAD5). Three start codons are used almost equally with ATP6, Cytb, COII and COIII are initiated with ATG, ATP8, COI, ND3, ND4L start with ATT, and ND1, ND2, ND4, ND5 and ND6 begin with ATA. The most frequent stop codon was TAA, while another stop codon TAG was used in COI, ND3, ND4L, and ND5. The length of 12S and 16S rRNA genes is 660 and 1,177 bp, respectively. The lengths of the 22 tRNA genes ranged from 55 (trnS2) to 68 bp (trnQ). Apart from 20 tRNA genes coding for 20 amino acids, 2 additional tRNA genes are observed for serine and leucine amino acids. All of the tRNA genes exhibited typical secondary structure except trnS1 and trnS2 which lacked a stable dihydrouridine arm loop.

A phylogenetic analysis was performed for 14 Copepoda species based on concatenated nucleotide alignment of 13 mitochondrial protein-coding genes and two rRNA genes using MAFFT alignment in Geneious 9.1.3 (Biomatters). A Maximum-likelihood tree was constructed using PhyML online server (http://www.atgc-montpellier.fr/phyml/, Guindon et al. 2010), using GTR + I + G substitution model with 1000 bootstrap replicates. The analysis showed that *C. simillimus* was grouped within *Calanus* genus with high bootstrap values and the five *Calanus* species form a monophyletic group (Figure 1). Given the ecological importance of *C. simillimus* in the Antarctic ecosystem and limited previous genetic information, the complete mitogenome of *C. simillimus* together with raw shotgun genomic sequences will be a useful resource for species identification and population genetics, as well as for phylogenetic and comparative genomics studies in *Calanus* and copepods.

**Figure 1.**
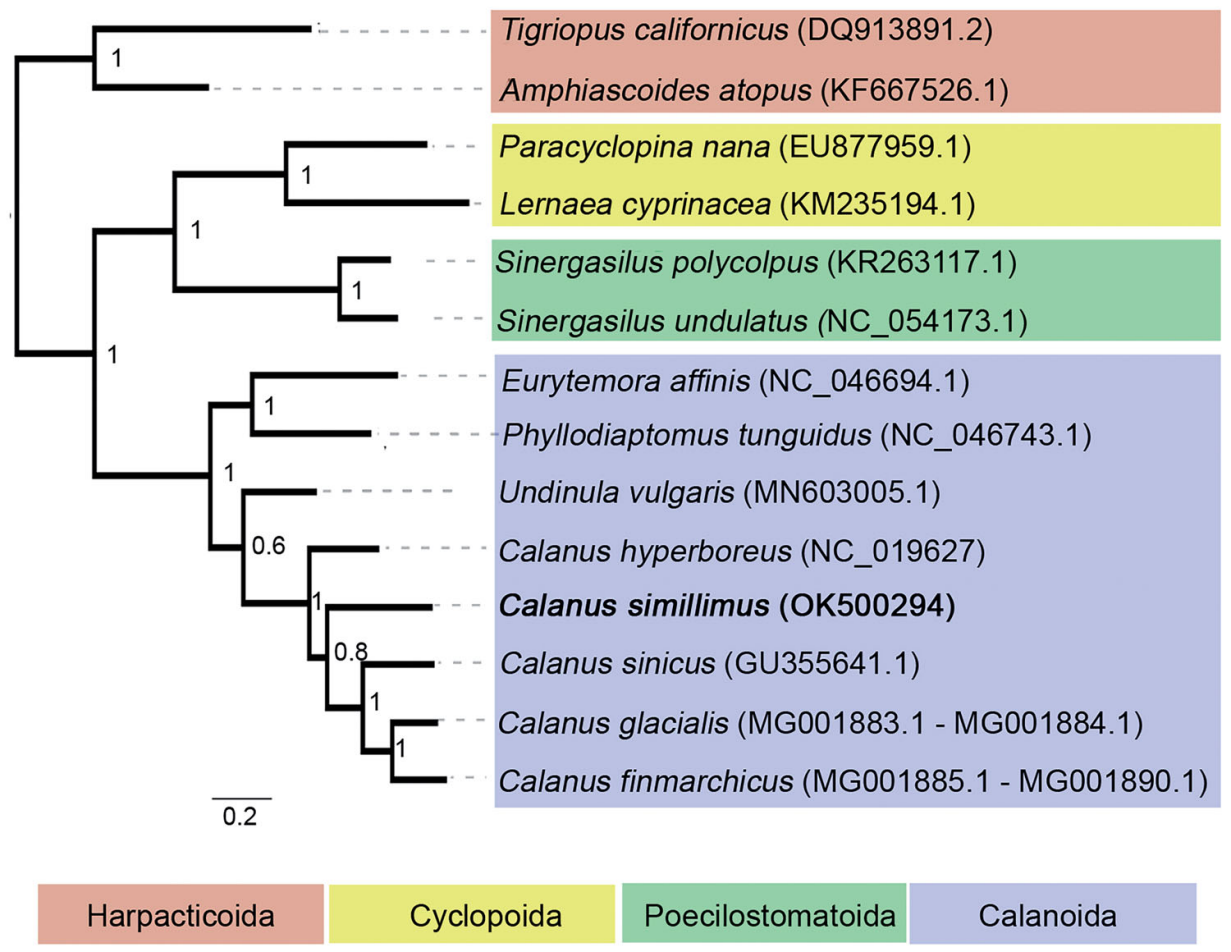
Maximum-likelihood tree of nucleotide alignment of 13 mitochondrial protein-coding genes and 2 rRNA genes in 14 copepod species. Order Harpacticoida is used as outgroup. *C. simillimus* investigated in this study is in bold. The number at each node is the bootstrap probability (1000 replicates).

## Data Availability

The genome sequence data that support the findings of this study are openly available in GenBank of NCBI at https://www.ncbi.nlm.nih.gov/ under the accession no. OK500294. The associated BioProject, BioSample, and SRA numbers are PRJNA769242, SAMN22106487, and SRR16229808 respectively.
